# Optimized culturing conditions for an algicidal bacterium *Pseudoalteromonas* sp. SP48 on harmful algal blooms caused by *Alexandrium tamarense*

**DOI:** 10.1002/mbo3.803

**Published:** 2019-02-07

**Authors:** Yi‐Hua Lyu, Yue‐Xia Zhou, Yi Li, Jin Zhou, Yi‐Xiao Xu

**Affiliations:** ^1^ South China Sea Environmental Monitoring Center State Oceanic Administration Guangzhou P.R. China; ^2^ Food and Drug Administration Linqing Shandong Province P.R. China; ^3^ School of life Science Henan Normal University Xinxiang Henan P.R. China; ^4^ Division of Ocean Science and Technology Graduate School at Shenzhen Tsinghua University Shenzhen Guangdong Province P.R. China; ^5^ Key Laboratory of Environment Change and Resources Use in Beibu Gulf Ministry of Education Guangxi Teachers Education University Nanning P.R. China

**Keywords:** *Alexandrium tamarense*, growth medium optimization, HABs control, optimum fermentation conditions, *Pseudoalteromonas *SP48

## Abstract

Bacteria play an important role in preventing algal blooms and reducing their harm to the environment. To improve the algicidal activity of *Pseudoalteromonas* SP48 which had an inhibition effect on dinoflagellate *Alexandrium tamarense*, its growth medium and fermentation conditions were optimized for this bacterium. In this study, we used two steps to establish the optimum conditions. First, the proper proportion of medium was selected based on an orthogonal design. Then, the fermentation conditions were further optimized through uniform design in an enlarged 5L bioreactor. To test the algicidal ability of *Pseudoalteromonas* SP48 under the optimum conditions, algal cell morphology was observed by transmission electron microscopy (TEM). After the orthogonal design, we found that the optimum medium was [0.7% (m/v) tryptone, 0.2% (m/v) soluble starch, 0.2% (m/v) sucrose, 0.1% (m/v) FeSO_4_, and 1.2% (m/v) K_2_ HPO_4_] for *Pseudoalteromonas* SP48 growth. Based on these results, optimum fermentation conditions were further explored in a 5L fermentation cylinder using a uniform design; the influence of variables such as incubation time, carbon type, and rotation speed were tested. The optimal fermentation conditions were fermentation time (42 hr), tryptone (1.1%), seeding volume (1.4 × 10^13^ cells), and rotation speed (250 r/min). Under these established optimum conditions, the biomass of strain SP48 increased by 79.2% and its lethal dose 50% (LD_50_) decreased by 54.0%, respectively. The TEM results showed that compared with the control group, the cell wall and cell membrane of *A. tamarense* were significantly damaged, and the structure and shape of the organelles were destroyed by algicidal bacteria of *Pseudoalteromonas* SP48. Overall, our results demonstrate that the optimized culture conditions could significantly enhance the algicidal activity of *Pseudoalteromonas* SP48 against a harmful dinoflagellate, such as *A*. *tamarense*. It will effectively provide a scientific foundation for both production of algicidal substances and HABs control.

## 1 INTRODUCTION

Harmful algal blooms (HABs) are caused by rapid propagation of algae under eutrophication and pose a significant threat to both environmental and human health (Anderson, 2009; Medlin, 2013). In recent years, HABs have frequently flourished alongside pollution deterioration and climate change (Li et al., 2014). The impact of HABs includes human intoxications resulting from ingestion of contaminated seafood, loss of wild or cultured fish resources, and impairment of tourism. In some cases, some high‐biomass HABs can also induce hypoxia or anoxia, killing marine organisms, and alterations marine trophic structure, all of which cause both major economic losses (Hickey et al., 2013) and ecological damage (Riegl, Bruckner, Samimi‐Namin, & Purkis, 2012). Harmful algae constantly introduce toxins into the environment; these toxins jeopardize marine organisms and destroy the stability of marine ecosystems (Mulvenna et al., 2012; Reichwaldt, Song, & Ghadouani, 2013). *Alexandrium tamarense*, a toxic marine dinoflagellate, can produce paralytic shellfish poison (PSP) causing death of marine organisms (Asakawa, Miyazawa, Takayama, & Noguchi, 1995; Cembella et al., 2002). PSP is the most widely distributed and damaging red‐tide toxin (Anderson et al., 1996; Kodama, Sato, Sakamoto, & Ogata, 1996; Lilly, Halanych, & Anderson, 2007), and has huge, negative impacts on marine fisheries and human health. Given its destructive effects, PSP has become a primary research issue, requiring urgent resolution (Ichimi, Suzuki, & Ito, 2002; Murata, Nagashima, & Taguchi, 2012; Suzuki, Ichimi, Oshima, & Kamiyama, 2003).

Several methods are used to regulate and control HABs; these measures include physical, chemical, and biological methods (Sengco & Anderson, 2004). Currently, high cost and low reliability of physical and chemical methods limit their application in HABs control (Zhou, Chen, & Zheng, 2010). Factors regulating red‐tide dynamics include algal‐bacterial interactions, which are increasingly cited as potential HAB regulators (Yang et al., 2012) and key components that perform major roles in regulating HAB dynamics (Bai, Huang, Su, Tian, & Zheng, 2011; Li et al., 2013; Wang et al., 2012; Zhang et al., 2013). *Pseudoalteromonas* sp. SP48 is a strain of bacterium that shows high algicidal activity on *A. tamarense*; it was isolated by our group from a red‐tide area in the Donghai Sea, China. After algicidal experiments, results revealed that bacterium SP48 indirectly attacked *A*. *tamarense* cells (Su et al., 2007). Algicidal activity of strain SP48 is dependent on the concentration of the algicidal compound inoculated into *A*. *tamarense* cultures (Wang et al., 2010). Unknown algicidal compounds may be metabolic byproducts of strain SP48, as previous reports found that *Pseudoalteromonas*, such as *Pseudoalteromonas* sp. T827/2B (Baker & Herson, 1978) and *Pseudoalteromonas* sp. A28 (Lee et al., 2000), secrete a variety of extracellular bioactive agents (Xi, Zhu, Liu, & Han, 2005). These agents show algicidal effects on algal species through indirect attack. To further improve the algicidal efficiency of strain SP48 and understand its characteristics and possible control mechanisms, this study investigated its effects of medium and various culture conditions on the algal growth.

Three types of experimental techniques have been used in the optimization of the microbial culture media: single‐factor test, orthogonal design, and uniform design. The present study utilized a combination of the single‐factor test and orthogonal design, which is a commonly utilized method in optimization of culture media. In contrast, uniform design is an experimental design strategy for statistical optimization of systems (Fang & Ma, 2001; Fang & Wang, 1996). This strategy allows for the largest possible number of culture or fermentation scales for each physicochemical factor and number of levels that can equal the number of experimental runs (Xia, Ji, & Wang, 2005). Therefore, uniform design is more advantageous when compared with other experimental statistical designs, such as orthogonal design, because it requires fewer trials (Zhang, 1995), and has been successfully applied in various field environments accordingly.

Based on these characteristics, here, the algicidal effect and growth conditions of algicidal bacterium *Pseudoalteromonas* SP48 were optimized by orthogonal design and regression analysis. The aim of this study was to obtain more efficient microbial method to inhibit harmful algae. The final objective was to provide useful information for future HAB management in the marine environment at laboratory or field scale.

## 2 MATERIALS AND METHODS

### 2.1 Algal cultures and algicidal bacterium

*Alexandrium tamarense* ATGD98‐006 was supplied by the Algal Culture Collection, Institute of Hydrobiology, Jinan University, Guangzhou, China. The *A*. *tamarense* strain was maintained in f/2 medium prepared with natural seawater (Guillard, 1975) at 20 ± 1°C under a 12:12 hr light‐dark cycle with light intensity of 50 μmol photons m^−2^∙s^−1^. After repeated washing by the medium, which was incorporated with lysozyme/sodium dodecyl sulfate and antibiotic treatment [a mixture of gentamycin (50 μg/ml), streptomycin (25 μg/ml), cephalothin (10 μg/ml), and rifampicin (5 μg/ml)], *A*. *tamarense* cultures were observed as axenic.

*Pseudoalteromonas* sp. SP48 is a bacterial strain that is thought as an efficient algicidal to *A. tamarense*; it was isolated from the red‐tide area in Donghai Sea, China (Su et al., 2007) and was stored at −80°C in 2216E medium (5 g peptone, 1 g yeast extract, 0.1 g FePO_4_ in 1L natural seawater, and pH 7.0–7.8) supplemented with 10% (v/v) glycerol. Strain SP48 was cultured in 2216E medium for 12 hr at 28°C with shaking at 150 r/min. The strain SP48 was deposited in State Oceanic Administration (SOA) of China with a store number of MCCC1K02697‐SP48.

### 2.2 Analysis of algicidal rate

Algicidal rate was calculated according to Equation (1) (Zhang et al., 2016):

(1) $$\text{Algicidal rate}\;\left(\%\right)=\left(N_{C}-N_{E}\right)/N_{C}\times 100$$

where *N*_*C*_ and *N*_*E*_ represent the number of algal cells in control and experimental groups, respectively. Algicidal rate of samples from an algal inoculation system was determined every 12 hr. Algal cells were counted after fixing with Lugol's iodine reagent. LD_50_ (lethal dose 50%) was used as a measure of the algicidal rate of strain SP48 (Zhang et al., 2016).

(2) $${\text{LD}}_{50}\left(\%\right)=\frac{\text{Vb}}{\text{Va}}\times 100$$

where *Vb* and *Va* represent the volume of bacteria and algal incubated respectively. All experiments were repeated in three biological replicates. The control group consisted of normal‐growth algae while adding sterile 2216E or sterile f/2 medium to avoid medium influence.

### 2.3 Optimization of culture conditions on strain SP48

To test the influence of different culture times on algicidal activity, bacterial culture was added to 50 ml 2216E medium with 1.0% concentration (bacterium, v/v) and was cultured under 28°C with shaking at 150 r/min. Samples were collected every 2 hr from 0 to 20 hr. Experimental groups were cultured at 20, 25, 28, 30, and 35°C to study the effects of temperature on the growth and algicidal effect of strain SP48. Initial pH values of 5.0, 6.0, 7.0, 8.0, 9.0, and 10.0 were used to study the effect of pH on growth and algicidal activity. Salinity levels in different experiment groups were set to 10, 20, 30, 40, and 50‰ in 2216E medium under 28°C to test the influence of salinity on algicidal effect. In different experimental groups, influence of rotation speed on algicidal effect was tested at 120, 150, 180, and 210 r/min in 2216E medium under 28°C for 24 hr. Each experimental group was sampled every 24 hr in duplicate. Three samples were used to measure absorbance value at a wavelength of 600 nm to indicate the growth rate of bacterium. After incubation, these bacterial samples (from the above‐mentioned optimized conditionsand during the exponential growth phase) were added into algal cultures to investigate algicidal rate.

### 2.4 Optimization of medium components on strain SP48

In order to optimize the medium components on bacterium SP48, we used the basal medium (2216E excluded nitrogen and carbon sources) to test the growth and algicidal activity effect of different nitrogen, carbon sources and mineral substrates. Peptone, tryptone, beef extract, yeast extract, and soybean peptone were added to the basal medium (0.1 g ferric phosphorous acid, and pH 7.0 in 1L natural seawater) to determine the optimum nitrogen source. Soluble starch, sucrose, and glucose were used as different carbon sources, and added to the basal medium with the determined optimum nitrogen source to determine optimum carbon source. To investigate effects of K_2_HPO_4_, FeSO_4_, MgCl_2_, and CaCl_2_ on growth and algicidal effect of strain SP48, different minerals were added to the Tryptone‐NaCl medium (here, the tryptone was selected, because it was the optimum N source in basal medium). The concentrations of K_2_HPO_4_, FeSO_4_, MgCl_2_, and CaCl_2_ in medium were 5.0 mg/L, 3.0 mg/L, 0.2 mg/L and 0.1 mg/L, respectively. Six different cultures were analyzed every 24 hr. Three samples were used to measure absorbance value at a wavelength of 600 nm, to determine the growth rate of bacterium. Other samples were used to investigate algicidal rate. The biochemical reagents (analysis grade, purity ≥ 99.9%) used in this study were purchased from Shanghai Chemical Reagent Co., Ltd (Shanghai, China).

### 2.5 Orthogonal design

Based on a single‐factor test and relative high cost performance as well as facility, tryptone, soluble starch, sucrose, FeSO_4_, and K_2_HPO_4_ were selected for the orthogonal design. To examine interactions among nutritional components of the culture medium, and to optimize their concentrations for production of algicidal substances, L_l6_ (4^5^) orthogonal arrays were used. Table 1 shows factor and level assignments of each factor. Based on a L_l6_ (4^5^) orthogonal array design, we performed 16 experiments in triplicate (Table 2).

**Table 1:** Levels of orthogonal design

Levels	Tryptone (A, %)	Soluble starch (B, %)	Sucrose (C, %)	FeSO_4_(D, %)	K_2_HPO_4_ (E, %)
1	0.3	0.05	0.05	0.05	0.4
2	0.5	0.1	0.1	0.1	0.8
3	0.7	0.2	0.2	0.15	1.2
4	1.0	0.4	0.3	0.2	1.6

**Table 2:** Orthogonal design and algicidal results

Experiment no.	Tryptone (A, %)	Soluble starch (B, %)	Sucrose (C, %)	FeSO_4_ (D, %)	K_2_HPO_4_ (E, %)	Algicidal activityLD_50_ (%)
1	0.5	0.1	0.1	0.1	0.4	6.378
2	0.5	0.4	0.2	0.05	1.2	4.460
3	0.3	0.1	0.2	0.2	0.8	8.501
4	0.7	0.2	0.2	0.15	0.4	5.161
5	0.3	0.05	0.05	0.05	0.4	16.867
6	0.3	0.4	0.1	0.15	1.6	12.005
7	0.7	0.05	0.1	0.2	1.2	4.268
8	0.3	0.2	0.3	0.1	1.2	4.705
9	1.0	0.2	0.1	0.05	0.8	4.766
10	0.5	0.2	0.05	0.2	1.6	6.093
11	0.7	0.4	0.05	0.1	0.8	0.948
12	0.5	0.05	0.3	0.15	0.8	12.588
13	1.0	0.5	0.2	0.1	1.6	6.546
14	0.7	0.1	0.3	0.05	1.6	5.944
15	1.0	0.4	0.3	0.2	0.4	3.913
16	1.0	0.1	0.05	0.15	1.2	2.338
K1	42.08	40.27	26.25	32.04	32.32	
K2	29.52	23.16	27.42	18.58	26.80	
K3	16.32	20.73	24.67	32.09	15.77	
K4	17.56	21.33	27.15	22.78	30.59	
1	10.519	10.067	6.562	8.009	8.080	
2	7.380	5.791	6.855	4.645	6.701	
3	4.081	5.181	6.167	8.023	3.943	
4	4.391	5.332	6.788	5.694	7.647	
R	6.439	4.886	0.688	3.738	4.137	
Factor order	A > B > E > D > C					
Optimal level	3	3	3	2	3	
Optimum combination	A3 B3 C3 D2 E3					

### 2.6 Uniform design

Uniform design was applied to determine optimum fermentation conditions for *Pseudoalteromonas* sp. SP48. For uniform design and subsequent analysis, data processing system (DPS, Version 3.01) software was used to generate experimental designs, statistical analysis, and the regression model (Tang & Feng, 1997). Experiment for optimization of fermentation conditions involved four factors, including culture time (h, X_1_), tryptone concentration (%, w/v, X_2_), seeding volume (cells, X_3_), and rotation speed (r/min, X_4_). Levels for each factor were selected depending on the experimental results of a single‐factor test. Uniform design table U_12_ (12^4^) was used for arrangement of 12 experiments (Table 3). Response evaluated (Y) was the dry weight of *Pseudoalteromonas* sp. SP48.

**Table 3:** Uniform design table U_12_ (12^4^) and the results

Experiment no.	X1	X2	X3	X4	Y
1	3 (18)	1 (0.3)	3 (4.2)	4 (250)	1.86
2	12 (72)	3 (0.7)	3 (4.2)	2 (150)	2.58
3	8 (48)	1 (0.3)	2 (2.8)	2 (150)	2.08
4	7 (42)	5 (1.1)	1 (1.4)	4 (250)	4.26
5	1 (6)	2 (0.5)	4 (5.6)	1 (100)	2.19
6	9 (54)	4 (0.9)	4 (5.6)	4 (250)	3.10
7	11 (66)	2 (0.5)	1 (1.4)	3 (200)	2.43
8	4 (24)	4 (0.9)	1 (1.4)	1 (100)	1.65
9	2 (12)	5 (1.1)	4 (5.6)	2 (150)	2.32
10	10 (60)	6 (1.3)	2 (2.8)	1 (100)	2.95
11	5 (30)	6 (1.3)	3 (4.2)	3 (200)	2.29
12	6 (36)	3 (0.7)	2 (2.8)	3 (200)	1.23

### 2.7 Algicidal activity measurement under orthogonal design

Algal cells were incubated with algicidal bacterium *Pseudoalteromonas* sp. SP48 after being optimized by orthogonal design for 24 hr, and then the lethal dose and TEM were investigated. Samples were fixed overnight at 4°C in 0.1 M phosphate‐buffered saline (PBS, 8 g NaCl, 0.2 g KCl, 1.44 g Na_2_HPO_4_, 0.24 g KH_2_PO_4_, in 1 L distilled water, 50 mM, and pH 7.4) containing 2.5% glutaraldehyde (v/v) and then post‐fixed in 1.0% OsO_4_ in the same buffer for 2 hr. After washing twice with PBS, samples were embedded in araldite resin. Sections (60–80 nm) obtained using an ultramicrotome were stained in 3.0% acetic acid/uranium‐citric acid and were viewed using a transmission electron microscope (model JEM‐2100HC; JEOL).

### 2.8 Biological assays

OD_600_ and bacterial dry weight were measured to reflect growth or biomass of *Pseudoalteromonas* sp. SP48. Algicidal rate and LD_50_ were determined to estimate algicidal activity of this bacterium on *A. tamarense*.

### 2.9 Statistical analysis

The data were processed by a one‐way analysis of variance (ANOVA) using SPSS version 13.0 (SPSS, USA). The average value of three replicate samples was expressed as mean ± *SD*. A value of *p* < 0.05 was considered statistically significant.

## 3 RESULTS AND DISCUSSION

### 3.1 Optimization of culture conditions for strain SP48

**Figure 1:**
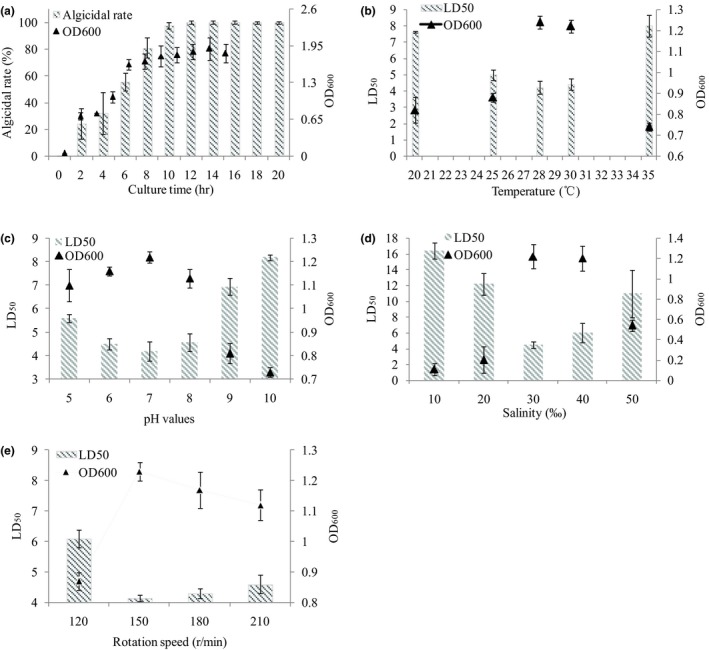
Influence of different culture conditions on strain SP48 growth and algicidal effect. Effects of culture time (a), temperature (b), initial pH (c), salinity (d) and rotation speed (e) on algicidal rate of *Pseudoalteromonas* sp. SP48. Data represent the mean + *SD* of triplicate measurements (*n* = 3)

Bacterial growth was significantly influenced by culture conditions, such as culture time, temperature, salinity, pH, and rotation speed during bacterial culture (Li et al., 2013). Figure 1 shows the optimization conditions for the *Pseudoalteromonas* sp. SP48 growth rate and algicidal activity under different culture environments. We speculated that with the biomass increase of strain SP48, the potential algicidal substances were secreted into extracellular components during culturing, and algicidal rate increased (*p* = 0.035) rapidly at the exponential phase (from 25% at 2 hr to almost 100% at 10 hr) (Figure 1a). Algicidal rate did not increase after the bacteria reached stationary phase. Therefore, stationary phase was the optimal culture time, and for strain SP48, culture time of >12 hr may allow bacterial cells to reach stationary phase. Temperature was the key factor governing bacterial growth and accumulation of metabolites. Growth rate and algicidal activity of strain SP48 were significantly different (*p* = 0.042) under varied culture temperatures (Figure 1b). When the strain was cultured at 28°C, OD_600_ reached the highest level (maximum biomass), and LD_50_ had the lowest levels (the highest algicidal activity). Results in Figure 1c show that LD_50_ of strain SP48 initially decreased, but then significantly increased when bacterial cells were cultured at different pH values (*p* = 0.031). Bacterial growth and algicidal activity reached their highest levels when strain SP48 was cultured at pH 7.0. Thus, optimal initial pH was 7.0. Strain SP48 was isolated from the marine environment; therefore, salinity is thought to play an important role in growth and algicidal substance production of *Pseudoalteromonas* sp. SP48 (Lin, Wang, Li, & Pan, 2011). When the medium salinity was 10‰, bacterial cells grew much slower and algicidal rate exhibited lower than that in other salinity levels. As salinity increased to 30 or 40‰, *Pseudoalteromonas* sp. SP48 showed an improvement in growth and algicidal activity. However, the growth and algicidal activity decreased again when salinity was >40‰ (Figure 1d). Therefore, optimal salinity was 30‰. An increase in rotation speed can provide more dissolved oxygen for culture media, which is helpful to the bacterial cell growth (Liu et al., 2011). Figure 1e shows that when rotation speed was lower than 150 r/min, LD_50_ value was high; but when rotation speed was higher than 150 r/min, LD_50_ value also increased. At 150 r/min, the LD_50_ value reached the lowest level, and the growth reached the highest level (*p* = 0.026). Thus, optimal rotation speed was 150 r/min.

### 3.2 Optimization of medium components for strain SP48

**Figure 2:**
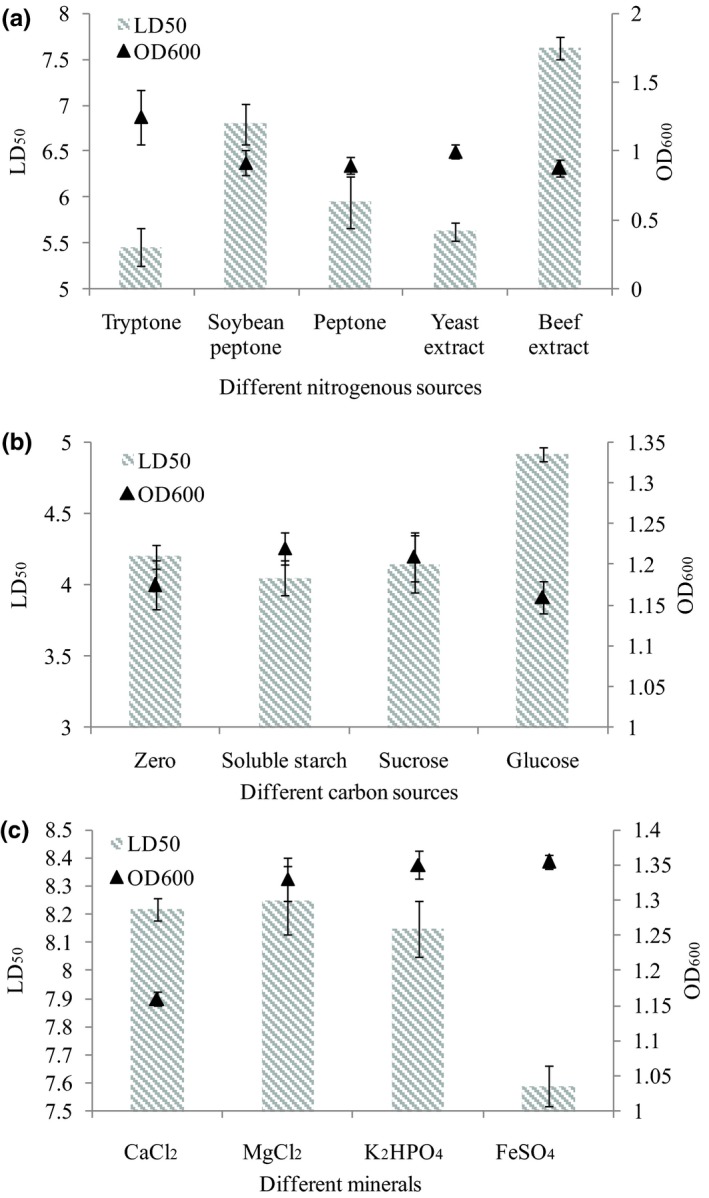
Influence of different medium components on strain SP48 growth and algicidal effect. Effects of nitrogen sources (a), carbon sources (b) and minerals (c) on algicidal activity of *Pseudoalteromonas* sp. SP48. (zero represents medium without carbon sources added). Data represent the mean + *SD* of triplicate measurements (*n* = 3)

To improve algicidal activity of strain SP48, we optimized the medium components. Figure 2 shows the results of optimization for bacterial growth rate and algicidal activity using different medium components (nitrogen, carbon, and minerals). Nitrogen is an essential nutrient factor for marine bacteria, and heterotrophic bacteria need to obtain nitrogen from additional sources for normal growth. In the present study, different nitrogen sources produced significantly different effects on growth and algicidal activity of strain SP48 (Figure 2a). Compared with other nitrogen sources, tryptone led to the highest OD_600_ and the lowest LD_50_ values (*p* = 0.011); thus, tryptone is an optimal nitrogen source for bacterial biomass and algicidal activity. Rashid, Rao, and Kornberg (2000) also reported that tryptone is an important nitrogen source for bacterial growth. Carbon source and minerals are also essential to microorganisms and help cells maintain the osmotic pressure vital for their growth and division (Her & Huang, 1995; Pósfai, Buseck, Bazylinski, & Frankel, 1998). Figure 2b shows that soluble starch and sucrose promoted the growth and algicidal level of strain SP48, and the former was the optimum carbon source. We speculate that soluble starch is the optimal carbon source due to its higher chemical oxygen demand (COD), which leads to a higher growth as they have a higher number of electron donors available for the bacterium (He, Minteer, & Angenent, 2005). In addition, the more degradable radio of soluble starch has also contribute to it. However, to determine the ‘best substrate’, a normalized analysis (based on COD) is required in future. Minerals are integral components for cell growth and metabolism of microorganisms, and different minerals have various effects on cell growth and the bioactive agent production of bacteria. Here, we determined the most suitable minerals for strain SP48 by comparing four minerals (CaCl_2_, MgCl_2_, K_2_HPO_4_, and FeSO_4_). Results in Figure 2c indicate that bacterial growth was promoted by all minerals except CaCl_2_. Compared with other minerals, FeSO_4_ induced a significant increase in bacterial growth rate and algicidal activity (*p* = 0.036). The possible reason was that iron can be acted as a common enzyme co‐factor, and help bacterial cell regulate osmotic gradient better (Argandoña et al., 2010).

### 3.3 Orthogonal design for optimum culture medium of strain SP48

Culture medium of *Pseudoalteromonas* sp. SP48 was optimized using orthogonal design, and results are shown in Table 2. Table 2 provides variance analysis for experimental results and optimal levels of each factor for obtaining lower LD_50_ values. Factor effects on LD_50_ follow: tryptone > soluble starch > K_2_HPO_4_ > FeSO_4_ > sucrose; the optimum conditions follow: 0.7% tryptone, 0.2% soluble starch, 0.2% sucrose, 0.1% FeSO_4_, and 1.2% K_2_HPO_4_.

### 3.4 Uniform design for optimum fermentation conditions of strain SP48

Results of uniform design are shown in Table 3. Bacterial dry weight varied considerably from 1.23 to 4.26 g/L^−1^ under different fermentation conditions. Regression analyses of data were performed using DPS software, and the regression equation obtained is as follows:

(3) $$Y=-0.759+0.119\times X_{1}-2.141\times X_{3}-0.000375\times X_{1}X_{1}+0.0312\times X_{2}X_{2}+2.193\times X_{3}X_{3}+0.0000322\times X_{4}X_{4}-0.00408\times X_{1}X_{2}-0.000175\times X_{1}X_{4}-0.246\times X_{2}X_{3}-0.00365\times X_{3}X_{4}$$

where the evaluated response *Y* is the dry weight of *Pseudoalteromonas* sp. SP48, and *X*_1_, *X*_2_, *X*_3_, and *X*_4_ represent factors of fermentation time (hr), tryptone concentration (%), seeding volume (10^13^ cells), and rotation speed (r/min), respectively.

Analysis of variance (ANOVA) results for a quadratic polynomial model strongly support the model, with a high model *F*‐value (46377.66) and a low *p* value (0.0036). High *R* ^2^ values (0.9999) indicate a strong relationship between experimental and predicted values in this experiment. The value of *R* ^2^ (0.9999) suggests that 99.99% of the total variation in the optimization of fermentation conditions is attributable to the independent variables and that the model is only unable to explain approximately 0.01% of the total variation. So the model is suitable for describing the relationship between fermentation optimization condition and significant factors.

The largest bacterial dry weight (4.26 g/L) was obtained when major factors were as follows: 1.1% tryptone, 1.4 × 10^13^ cells seeding volume, 42 hr fermentation time and 250 r/min rotation speed. A verification test was performed, and bacterial dry weight reached 4.58 g/L under optimal fermentation conditions. A relative error of 0.77% was predicted between the verification test and the regression model, demonstrating that the uniform design can be used for optimization of fermentation conditions. Interestingly, the optimum agitation value in the laboratory experiment and the uniform experiment were 150 and 250 rmp, respectively. A possible reason for this maybe because the rpm value was tested at culture conditions that were different from the determined optimum values, and also caused by the fermentation magnification effect (Zhang & Chu, 2003).

### 3.5 Algicidal effect and algicidal procedure of strain SP48 with optimized medium

**Figure 3:**
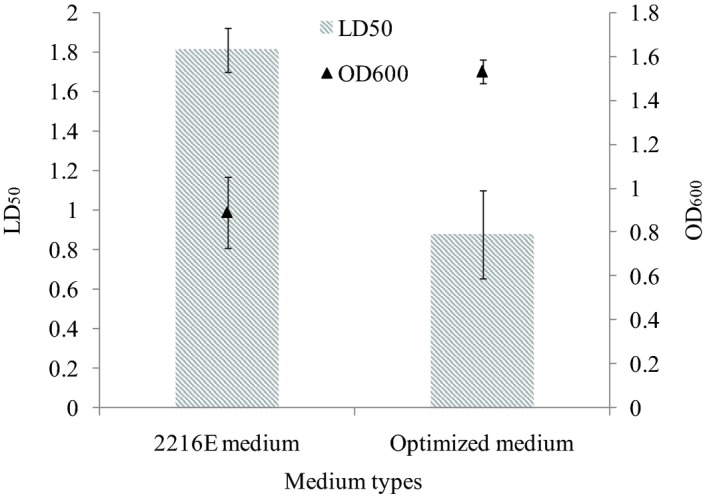
Results of biomass and algicidal activity of strain SP48 with 2,216 medium and optimized medium. Data represent the mean + *SD* of triplicate measurements (*n* = 3)

Based on previous experimental results, a comparison between the 2216E medium and the optimized medium was conducted using the algicidal effect and biomass of strain SP48. Figure 3 shows that OD_600_ of bacterium increased by 79.2%, whereas algicidal effect (LD_50_) reached 0.76%, indicating a decline of 54.0%. Holmström and Kjelleberg (1999) studied the production of biologically active extracellular agents in several *Pseudoalteromonas* species and found they were nutrient dependent. The cells did not express any antibacterial activity and the production of active compounds was very low when *Pseudoalteromonas* species were grown with unsuitable nutrients (Gauthier, 1976). Hence, suitable, available nutrient conditions are essential for the bacteria to express their extracellular biological active compounds. This study ensured the optimal medium and fermentation conditions for strain SP48, a high yield of the algicidal substances were obtained, and algicidal activity remained high in the optimized medium.

**Figure 4:**
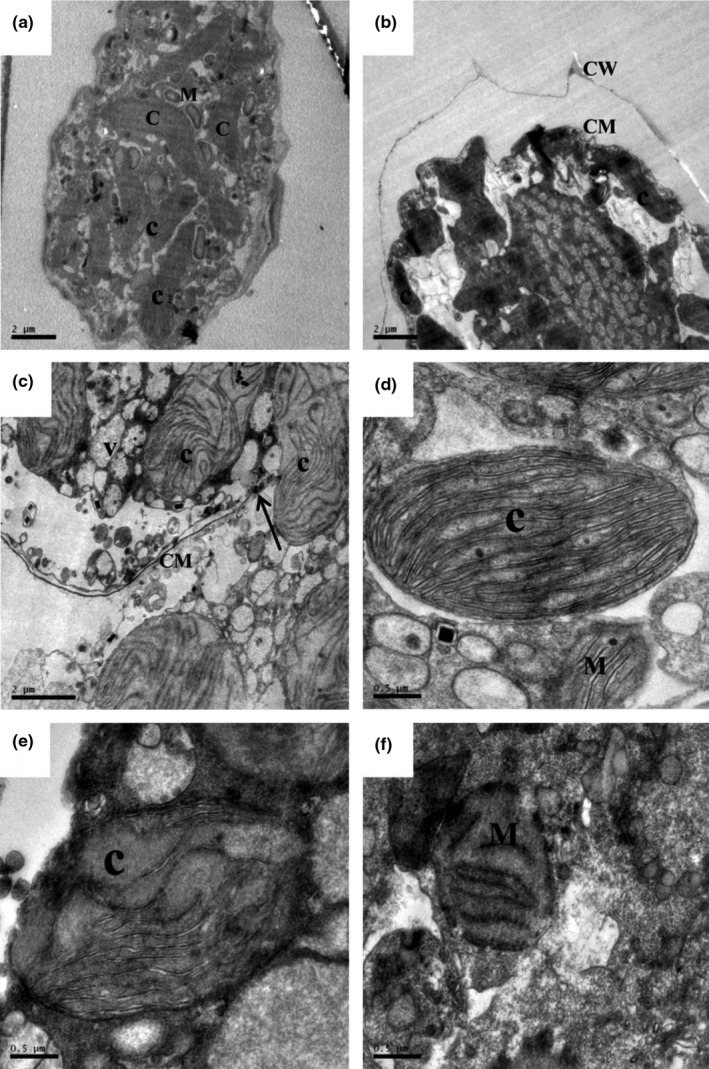
Transmission electron micrographs of *Alexandrium tamarense* lysis under the effect of strain SP48 with optimized medium. (a and d) Control cells and cells with chloroplasts and mitochondria in *A. tamarense*; (b) damaged *A. tamarense* cells after 12 hr treatment; (c) damaged *A. tamarense* cells after 24 hr treatment; arrow shows where cell wall and cell membrane were broken; (e) damaged chloroplast after 24 hr treatment; (f) damaged mitochondrion after 24 hr treatment. (C: chloroplast; m: mitochondria; CM: cell membrane; CW: cell wall; V: vacuole). Bars (a), (b) and (c) 2 μm; (d), (e) and (f) 0.5 μm

Algicidal effect produced by strain SP48 was observed with TEM (Figure 4). Control cells in Figure 4a show dense multi‐lobed intact chloroplasts, mitochondria, as well as cytoplasm, cell walls and cell membranes that have been kept intact. After treatment with strain SP48 for 12 hr, morphological characteristics and even structural damage were found in algal cells in the treatment group (Figure 4b). Distinct plasmolysis was observed, and organelles were blurred. Within 24 hr of treatment of strain SP48, cell membrane integrity and the structure of organelles were seriously damaged. Cytoplasm and organelles spilled out of cells, and the number of multi‐vesicular bodies increased significantly (Figure 4c). Figure 4d–f provides a detailed view of chloroplast structure destruction. Compared with that of normal cells without exposure to strain SP48, the algicidal effect was to cause severe damage to the chloroplast structures, lose the organelle membrane integrity significantly, and induce the thylakoid outflow. Cell membrane integrity and cell structure intactness are essential to algal cell growth. However, the cell wall and cell membrane treated with algicidal bacteria SP48 were significantly damaged, and the structure and shape of organelles were simultaneously changed. Zhang et al. (2013) also reported that algal cell lysis was observed under algicidal effect of bacterium BS01.

Using *Pseudoalteromonas* as a algicidal substance for harmful algal blooms has good prospects, though further study need to be done for the evaluation of its wide development and industrial production. Currently, only few reports provide information on this genus and on the relationship between bacteria and algae species which can cause harmful algal blooms. Based on this study, next we will isolate these algicidal substances and identify their molecular structure. Such work has already been underway in our laboratory, and hopefully it will help us to develop new algicidal substances for the mitigation of HABs.

## CONFLICT OF INTEREST

The authors declare that they have no conflict of interest.

## AUTHORS CONTRIBUTION

YXZ carried out strain isolation and related experiment. YHL and YXZ drafted the manuscript, YL and YXX supervised statistical analysis. JZ and YXX contributed to experimental design, comment, funding support and approved the final manuscript.

## ETHICS STATEMENT

None required.

## Data Availability

All data are provided in full in the results section of this paper.
